# The reflective measurement model of adherence to non-pharmaceutical interventions (NPIs) in accordance with normalization process theory (NPT) in coherent and convenient social subgroups: PLS-SEM analysis

**DOI:** 10.1093/eurpub/ckae085

**Published:** 2024-05-09

**Authors:** Magda Pletikosa Pavic, Shelly Melissa Pranic, Tonci Mastelic, Zeljko Kljucevic, Majda Gotovac, Anamarija Jurcev Savicevic, Tonci Kozina, Slavica Kozina

**Affiliations:** Department of Mental Health, Split-Dalmatia County Teaching Institute of Public Health, Split, Croatia; Department of Public Health, University of Split School of Medicine, Split, Croatia; Department of Psychiatry, University Hospital Split, Split, Croatia; Department of Mental Health, Split-Dalmatia County Teaching Institute of Public Health, Split, Croatia; Department of Mental Health, Split-Dalmatia County Teaching Institute of Public Health, Split, Croatia; Department of Public Health, University of Split School of Medicine, Split, Croatia; Department of Respiratory Disease Prevention, Split-Dalmatia County Teaching Institute of Public Health, Split, Croatia; University Department of Health Studies, University of Split, Split, Croatia; Department of Professional Studies, University of Split, Split, Croatia; Department of Medical Psychology, University of Split School of Medicine, Split, Croatia

## Abstract

**Background:**

Non-pharmaceutical interventions (NPIs) decrease COVID-19 transmission. Reliability and validity of adherence to NPIs in accordance with normalization process theory (NPT) in coherent and convenient social subgroups using reflective measurement model assessment has not been evaluated.

**Methods:**

In February 2021, a sample of medical students and people with substance use disorders in treatment as coherent (based on continuous probability distribution) vs. convenient groups (based on convenience, not equal probability) composed of travellers and COVID-19 suspected persons from Split-Dalmatia County (SDC) (*n* = 656) in the Mediterranean completed self-administered surveys. Partial least squares structural equation modelling (PLS-SEM) was used to measure reflective model assessment of adherence to NPIs according to NPT.

**Results:**

PLS-SEM reflective model assessment provided two-group specific factors in inverse relationships which determined adherence to NPIs with excellent goodness-of-fit [*χ*^2^ = 1.292, df = 1; *P* = 0.297, CFI = 1, TLI = 0.997, RMSEA = 0.011 (90% CI 0–0.105), RMSEA *P* = 0.604, SRMR = 0.008, Hoelter CN (*α* = 0.05) = 2322.757]. Significant negative factors covariance estimate (−0.716) revealed an inverse relationship between first (adherence to NPIs and internal locus of control (LoC) (0.640)) and second factor; young adulthood age (≤25) and highest level of education (1362). As the first factor increased the second tended to decrease. LoC is expected potential mechanism by which sex (ML_sex_ = −0.017, SE = 0.007, *P* < 0.016) and belonging to coherent subgroups (ML_group_ = −0.008, SE = 0.003, *P* = 0.015) can produce indirect effect of adherence to NPIs.

**Conclusions:**

Coherent subgroups had a more pronounced tendency toward integration of NPIs in everyday life. Group factors that facilitate the normalization were higher educated younger adults with a tendency toward external LoC.

## Introduction

The impact of the COVID-19 pandemic, declared by the WHO on 11 March 2020, affected diverse populations worldwide.^1^ Non-pharmaceutical interventions (NPIs), like social distancing, wearing masks and hand hygiene, were implemented worldwide to curb the spread of SARS-CoV-2.^2^ While COVID-19 is not the first pandemic, neither is use of NPIs in the prevention of infectious diseases.

Historically, NPIs have played a crucial role in public health. In 1377, the Government of the Republic of Dubrovnik (Croatia) enacted laws prioritizing public health over private interests, introducing the motto ‘OBLITI PRIVATORUM—PUBLICA CURATE’ (‘Forget the private—take care of the state’), they strived for hygiene and overall health. The concept of quarantine, isolating the sick and restricting movement to prevent disease spread, dates back to this period where ‘plague’ or ‘pestilence’ was used to describe all infectious diseases.^3^ Dubrovnik’s initiative inspired establishment of quarantine systems such as in 1581 in Split (Croatia), a significant sanitary cordon along the Ottoman Empire border in the 18th century, and later becoming a global measure to combat infectious diseases.^4^

Masks have a long history, with early mentions in 15th-century Iran as ‘panam’ or ‘pandam’,^5^ used during religious ceremonies to protect the sacred fire from breath's perceived impurities. In Pahlavi texts, priests wore veils resembling today's surgical masks to cover the face during ceremonies.^5^^,^^6^ Ancient Iranian doctors also used face coverings to prevent contamination while treating patients, a practice that endured through the Middle Ages. The significance of face masks in preventing respiratory virus transmission became apparent during epidemics and clinical practice. In 1897, Johann Mikulicz introduced face masks in operating rooms.^7^ Subsequently, during the Manchurian plague of 1910–1911 and influenza pandemics of 1918–1919, masks covering the mouth, nose and chin emerged as crucial tools for protecting medical staff and patients from infectious diseases beyond surgical settings.^7^^,^^8^

Ignác Fülöp Semmelweis (1818–1865), known as the ‘father of hand hygiene’, emphasized its importance, as did Florence Nightingale (1820–1910).^9^ In 1847, Semmelweis identified the cause of puerperal sepsis and mandated doctors to wash hands with chlorine, significantly reducing infections. Nightingale,^9^ during the Crimean War, implemented hand hygiene and hygiene measures, lowering the death rate from 42% to 2%. Despite their efforts, the practice was not widely accepted for over a century. In the fight against the COVID-19 pandemic, handwashing was a crucial NPI measure.

Adherence to NPIs varies based on the type of measures, individual and societal factors,^10^^,^^11^ or governmental regulations.^12^ During the Ebola outbreak in West Africa (2014–2015), NPIs were the only preventive measures available.^13^ Stringent measures, including a three-week village quarantine enforced by soldiers and police, demonstrated their historical effectiveness. Similar rigorous measures were implemented during the COVID-19 pandemic, as observed in a study in Brazil reporting an 87–93% reduction in the daily incidence trend of COVID-19 associated with NPIs.^14^ Deciding on quarantine implementation involves a delicate balance between health, safety, economic benefits and potential negative impacts on individual rights such as freedom of movement and choice. Factors influencing compliance with COVID-19 NPIs include individuals' beliefs in the inevitability of infection regardless of compliance [locus of control (LoC)]^11^ and perception of preventive measures as societal mandates (moral behaviour).^15^ The myriad of physical, psychological and social factors affecting adherence to NPIs makes establishing causality challenging, complicating the role of public health officials in prioritizing measures.^16^

Normalization process theory (NPT) is a valuable framework for studying and enhancing adoption and normalization of COVID-19 NPIs.^17^ NPT, a prominent social process theory, elucidates cognitive and social processes crucial for implementation within specific contexts.^18^ Applying NPT concepts allows tailored public health efforts, considering social and organizational contexts, leading to more effective implementation^19^ and sustained adherence to preventive measures like mask-wearing, social distancing and hygiene practices. NPT offers insights into roles of individuals, groups and organizations in promoting adherence to these measures within social norms, cultural factors and organizational structures.^17^^,^^19^^,^^20^

Our study employed NPT to understand factors influencing integration of NPIs into everyday life. Using PLS-SEM for reflective measurement assessment, we examined behavioural and group characteristics affecting adherence to NPIs. Adhering to NPT assumptions, our model considers group coherence, cognitive participation, reflexive monitoring and integration and normalization of previous behaviour during the COVID-19 pandemic. The study included medical students and people with substance use disorders as coherent groups, compared with a convenient group of travellers and those suspected of having COVID-19. Parameters such as LoC (an individual's belief about the extent to which they have control over the events in their life) and moral behaviour standards (compliance with social and moral norms) were considered as factors influencing capacity to adhere to epidemiological guidelines, along with sociodemographic variables (age, sex and education level).

The goal of this reflective measurement model assessment was to ensure reliability and validity of the construct of adherence to NPIs in accordance with NPT in coherent and convenient social subgroups using partial least squares structural equation modelling (PLS-SEM) analysis.

## Methods

We searched PubMed/MEDLINE using ‘normalization process theory’, ‘health care’ and ‘COVID-19’ search terms without language or other restrictions.

### Survey sample

The Ethics Committee of the Department of Mental Health at the Split-Dalmatia County (SDC) Teaching Institute of Public Health approved this study. All participants provided informed consent before completing questionnaires.

In this cross-sectional study conducted in Split, Croatia, a survey was administered via Google Forms in February 2021 to medical students (third to sixth year) and paper form to people with substance use disorders in outpatient treatment (coherent groups) and to convenient groups (travellers and those suspected of COVID-19). University of Split School of Medicine class representatives shared the survey link with students *via* WhatsApp. Paper surveys were distributed at COVID-19 testing sites at the SDC Teaching Institute for Public Health including both individuals suspected of infection and those who sought COVID-19 test results for travel and during outpatient counselling for substance use. We included adults aged ≥18 years residing in SDC.

### Sample size calculation and procedures

We used Raosoft sample size calculator with a 99% confidence level, 1%, margin of error and a response distribution of about 50% to determine a sample of 214 people suspected to be infected with COVID-19 from approximately 300 suspected infections per day, 102 travellers from approximately 110 people per day, 123 people with substance use disorders from approximately 25 people who underwent weekly therapy and 217 medical students from a total of 265 medical students from the third to sixth years.

### Survey instrument and measures

We assessed sociodemographic characteristics, personal LoC and behavioural parameters [Moral Behaviour Scale (MBS)] using a semi-structured questionnaire (15 questions) covering sex, age, education, previous COVID-19 infections, adherence to NPIs (see Supplementary Appendix A). Additionally, we utilized Rotter's LoC Scale,^21^ (29 statement pairs), to measure participants’ perceived control over their lives scoring between 0 and 23 points. Compliance with explicit social and moral norms was evaluated using 15 items from the MBS scale, modified by Mendez and colleagues,^22^ scoring between 15 and 60 points. Both scales represent a continuum between external and internal control and social–moral behaviour vs. social non-affirmative behaviour.

##  

### Data analysis

PLS-SEM assessed the reflective measurement model for adherence to NPIs in accordance with NPT. The model aimed to comprehend factors influencing integration of NPIs into the daily lives of distinct groups—cohesive (medical students and people with substance use disorders in treatment) and convenient (travellers and COVID-19 suspected individuals). Differences between characteristics such as sex,^23^ age (younger and older age),^24^ educational level (low and high),^23^ LoC,^25^ social behaviour,^26^ internal and external LoC and social–moral behaviour vs. social non-affirmative behaviour were explored.

We employed the PLS-SEM model to avoid circular relationships inherent in qualitative research, focusing on maximizing the unexplained variance in dependent measures (*R*^2^). The complexity of our PLS-SEM was evident as it is based on a different set of observed variables and model relationships. To meet PLS-SEM criteria,^27^ we aimed for a larger sample size to enhance estimation precision, and made no distributional assumptions, given the nonparametric nature of PLS-SEM. The scale of measurement for our variables differed from the metric data and quasi-metric scaled variables that PLS-SEM uses. We used dichotomous and categorical variables regarding adherence to NPIs (Yes/No), sex (M/F), age (18–25, 26–34, 35–44, 45 years and older), education level (elementary school, high school, bachelor’s degree or master’s degree) and LoC and MBS scores. Utilizing PLS-SEM for the reflective measurement of adherence to NPIs was essential to assess the construct validity of the model. Construct validity is crucial for testing the reliability, convergent validity and discriminant validity of measures.

First, we tested construct validity using SMART PLS (see repository^28^ for Supplementary Appendix B with the software-generated model).^29^ We used reflective measurement model statistics and empirical test criteria: indicator reliability (indicator loading), internal consistency reliability [Cronbach’s *α* and composite reliability (CR)], convergent validity [average variance extracted (AVE)], discriminant validity (Fornell–Larcker criterion, cross-loadings) and evaluation overview (all reflective and partial reflective criteria evaluated). Bootstrap procedures in PLS-SEM context were performed to determine the statistical significance of direct effects, non-direct effects, the coefficient of determination and the comparison of effects^30^ (see repository^28^ for Supplementary Appendix C with the software-generated model).

We addressed common method bias within the context of PLS-SEM, demonstrating the success of the full collinearity test in identifying such bias based on variance inflation factors (see repository^28^ for Supplementary Appendix B with the software-generated model).

Meaningfulness of considering endogeneity in PLS-SEM in explanatory modelling involved ‘use of statistical models for testing causal explanations’.^31^ Controlling for endogeneity is crucial to adequately test hypotheses. Endogeneity problems most often arise from omitted variables that correlate with one or more independent and dependent variable(s) in the regression model.^32^ We present the relationships between LoC, age, educational levels and adherence to NPIs (Yes/No as Bernoulli variables) using general linear modelling (GLM) (table 1 and Supplementary figure S1). It is important to emphasize that PLS-SEM application does not assess endogeneity. Bascle^33^ resolves the problem of endogeneity by decomposing endogenous independent variables’ variance into two parts. According to the literature,^33^^,^^34^ we decomposed endogenous independent variables (see Model 2 in tables 1 and 2 and Supplementary figure S1) into two parts (deemed two factors) in Model 3 (tables 1 and 2 and Supplementary figure S1—Model 3) and Model 4 (tables 1 and 2 and Supplementary figure S1—Model 4).

**Table 1:** Impact of locus of control, age and educational level on adherence to COVID-19 non-pharmaceutical interventions in adults in Split, Croatia in 2021

Predictors	Odds ratio (95% CI)
LoC	0.90 (0.85–0.95)
Age (years)	
Under 25	1 (Reference)
26–35	3.87 (2.13–7.10)
36–45	3.67 (2.12–6.46)
46 and older	4.63 (2.69–8.14)
Education level	
Elementary school	1 (Reference)
High school	0.38 (0.16–0.90)
Bachelor	0.29 (0.10–0.78)
Master	0.14 (0.06–0.33)

Note. CI = confidence interval.

Alt text: Table showing the influence of locus of control, age and educational level on adherence to COVID-19 non-pharmaceutical interventions in adults in Split, Croatia in 2021.

**Table 2:** Assessment of the generalized linear model fit for selecting an optimal model assessing psychological patterns and participant characteristics in adults in Split, Croatia, 2021^a^

				Baseline test			Difference test		
	AIC	BIC	*n*	*χ*²	df	*P*-value	Δ*χ*²	Δdf	*P*-value
Model 3	7965.099	8023.419	656	1.292	1	0.256			
Model 4	7964.892	8023.212	656	1.085	1	0.297	−0.206	0	
Model 2	7965.443	8019.277	656	3.636	2	0.162	2.551	1	0.110
Model 1	13222.014	13302.765	656	97.846	9	<0.001	94.210	7	<0.001

a AIC = Akaike’s information criterion; BIC = Bayesian information criterion; df = degrees of freedom.

Alt text: Table displaying generalized linear model fit information for selecting an optimal model assessing psychological patterns and participant characteristics in adults in Split, Croatia, 2021.

We refer to endogenous and exogenous to identify variables that endogeneity does (not/less) impact; we use dependent and independent to identify constructs—Model 2 (see tables 1 and 2 and Supplementary figure S1—Model 2) and Model 3 (tables 1 and 2 and Supplementary figure S1—Model 3) to explain our constructs of group factors that facilitate normalization and integration of NPIs into daily life in coherent and convenient groups—Model 4 (tables 1 and 2 and Supplementary figure S1—Model 4).

Considering the sensitivity of chi-square to sample size to assess model suitability in SEM, Alavi et al.^35^ proposed testing chi-square, root mean square error of approximation (RMSEA), comparative fit index (CFI > 0.90), standardized root mean squared residual (SRMR) combined and Tucker–Lewis index (TLI) > 0.90 to indicate acceptable model fit.^36^

Additionally, we conducted a comprehensive evaluation of Akaike’s information criterion (AIC), Bayesian information criterion (BIC) and their extensions for selecting an optimal model from the four models’ alternatives. We used the Kruskal–Wallis test to compare medians across four groups. Dwass–Steel–Critchlow–Fligner (DWCF) pairwise comparisons *post hoc* tests were used with the Kruskal–Wallis test to determine which specific comparisons were significant.

We reported frequencies, means ± standard deviation, medians with interquartile (IQR) range as well as 95% confidence intervals (CI). Odds ratios (OR) were reported along with 95% CIs. We used *jamovi* (version 2.2.5), JASP (version 0.18.3.0) (Windows 64 Bit) and Smart PLS 4 for statistical analysis, and considered a *P* < 0.05 as significant.

## Results

In this study conducted during the lockdown, 656 participants were recruited, comprising 32.6% (*n* = 214) COVID-19 suspected individuals, 15.6% (*n* = 102) travellers, 18.8% (*n* = 123) people with substance use disorders in treatment and 33.0% (*n* = 217) medical students. Participants’ ages ranged from 18 to 79 years (Supplementary table S1).

Regarding LoC and adherence to NPIs, the majority of women (*n* = 313/383, 82%) and men (*n* = 206/273, 75%) with an external LoC adhered to NPIs (Supplementary table S1). Specifically, participants aged 18–25 years (240/264) with an external LoC exhibited higher adherence. Conversely, participants aged 25–35 and those aged ≥45 years with an internal LOC tendency were less likely to adhere to NPIs (Supplementary table S1).

Participants holding master’s degrees (*n* = 302/656, 46%) exhibited a notable tendency toward external LoC and demonstrated high adherence to NPIs (Supplementary table S1). Medical students (*n* = 211/217, 97%) followed by people with substance use disorders (*n* = 66/123, 54%) had the most pronounced external LoC tendency (Supplementary table S1). There was a statistically significant difference in variance of adherence to NPIs according to sex, age, education and behaviours as explanatory variables (*P*-range = 0.001–0.05), with the most significant effect size of variance in age (*η*^2^ = 5.2%) and education (*η*^2^ = 5.0%), except for MBS (*P* = 0.711) (Supplementary table S2).

The DWCF test for the medians and variances of sex, age, education, internal vs. external LoC and MBS in adherence to NPIs showed specifically that all explanatory variables except moral behaviour (*P* = 0.711) significantly differed according to adherence to NPIs (*P* < 0.001) (Supplementary table S2). Participant age (*η*^2^ = 0.545 or 54.5%, *P* < 0.001) and their education level (*η*^2^ = 0.539 or 53.9%, *P* < 0.001) had the most significant effect on the sample variance of adherence to NPIs, other explanatory variables were significant (*P* < 0.001) with less impact (*η*^2^ = 0.04–0.175) (Supplementary table S3).

The DWCF test found a significant difference between medical students and all other groups (*P* < 0.001). The variances justify treatment of each group as homogeneous, especially medical students and people with substance use disorders (Supplementary table S4).^37^ According to AIC and BIC, Model 1 had an unsatisfactory fit index (*P* < 0.01) (tables 1 and 2 and Supplementary figure S1—Model 1). The most optimal path model was 4 (tables 1 and 2, and Supplementary figure S1—Model 4) where two factors including personal behaviour surrounding adherence to NPIs for COVID-19 had excellent goodness-of-fit **[***χ*^2^ = 1.085, df = 1; *P* = 0.297, CFI = 1, TLI = 0.997, RMSEA = 0.011 (90% CI 0.00–0.11), RMSEA *P* = 0.604, SRMR = 0.008 (Hoelter CN α = 0.05 = 2322.757)] (tables 1–3). The path coefficient between factors 1 and 2 was high (−716) and statistically significant (*P* < 0.001). There was a multilevel effect of the young adult age group (>18 and ≤25) and highest levels of education (bachelor’s and master’s degrees) that had a sizeable effect on the path coefficient for the association between adherence to NPIs and external LoC. The amount of unexplained variance in dependent measures for age (*R*^2^ = 0.452) and education (*R*^2^ = 0.391) can be considered substantial, because variances for dependent measures and LoC are greater than 0.200 and 0.109, respectively^38^ (tables 3 and Supplementary figure S1—Model 4).

**Table 3:** Goodness-of-fit indices of the partial least squares structural equation model assessing psychological patterns and participant characteristics in adults in Split, Croatia, 2021^a^

Index	Model 1	Model 2	Model 3	Model 4
Comparative fit index (CFI)	0.769	0.992	0.999	1.000
Tucker–Lewis index (TLI)	0.615	0.976	0.991	0.997
Information criteria				
Log-likelihood	−6593.007	−3970.721	−3969.549	−3969.446
Number of free parameters	18.000	12.000	13.000	13.000
Akaike information criterion (AIC)	13222.014	7965.443	7965.099	7964.892
Bayesian information criterion (BIC)	13302.765	8019.277	8023.419	8023.212
Sample-size adjusted Bayesian (SSABIC)	13245.615	7981.177	7982.144	7981.937
Other fit measures				
Root mean square error of approximation (RMSEA)	0.123	0.035	0.021	0.011
RMSEA 90% CI lower bound	0.101	0.000	0.000	0.000
RMSEA 90% CI upper bound	0.145	0.093	0.109	0.105
RMSEA *P*-value	2.904e − 8	0.575	0.565	0.604
Standardized root mean square residual (SRMR)	0.060	0.017	0.010	0.008
Hoelter's critical *N* (*α* = 0.05)	114.432	1081.913	1951.715	2322.757
Hoelter's critical *N* (*α* = 0.01)	146.258	1662.626	3370.239	4011.096
Goodness-of-fit index (GFI)	0.998	1.000	1.000	1.000
*R*-squared				
Adhere restrictions	0.106	0.127	0.127	0.200
Sex	0.170			
Age	0.447	0.445	0.384	0.452
Education level	0.408	0.388	0.292	0.391
Locus control LoC	0.090	0.073	0.067	0.109
Moral behaviour scale (MBS)	0.033			

Note. CI = confidence interval.

a Some models are based on a different set of observed variables.

Alt text: Table showing the goodness-of-fit indices of the partial least squares structural equation model assessing psychological patterns and participant characteristics in adults in Split, Croatia, 2021.

We used GLM to emphasize endogeneity, which arose from omitted variables (Model 2, tables 1 and 2 and Supplementary figure S1—Model 2) that correlate with one or more independent and dependent variable(s). We present relationships of adherence (0) or non-adherence^1^ to NPIs as a function of coherent vs. convenient groups’ characteristics (age and education) and psychological patterns of human behaviour (LoC), for which PLS-SEM analysis showed a significant effect: age (>18 and ≤25), higher levels of education and personal tendency toward external LoC were significant predictors of adherence to NPIs (*P* < 0.001) (table 4). Simply put, individuals older than 25 (>25 and ≥35) years were more likely to not adhere to recommendations than to adhere (table 4 and Supplementary figure S2). Higher education level than elementary was associated with a lower likelihood of non-adherence to NPIs (table 4 and Supplementary figure S2). An increase in LoC or tendency to external LoC was associated with a lower likelihood of non-adherence to NPIs (table 4 and Supplementary figure S2).

**Table 4:** Factor loadings of Model 4 for age, education level, locus of control and adherence to non-pharmaceutical interventions in an assessment of psychological patterns and participant characteristics in adults in Split, Croatia, 2021

Latent	Variable	Estimate	Std. error	*z*-Value	*P*	95% CI	All	LV	Endo
H2bF1	Age	1.000	0.000			1.000 to 1.000	0.673	0.800	0.673
	Education level	−0.779	0.127	−6.150	<0.001	−1.028 to −0.531	−0.625	−0.624	−0.625
H2bF2	LoC	1.000	0.000			1.000 to 1.000	0.330	1.167	0.330
	Adherence	−0.156	0.036	−4.343	<0.001	−0.226 to −0.085	−0.447	−0.182	−0.447

Note. H2bF1 = Factor 1 (Age and Education Level), H2bF2 = Factor 2 (Locus of control and Adherence), LoC = locus of control, LV = latent variable, Endo = endogenous variable.

Alt text: Table showing the factor loadings of Model 4 for age, education level, locus of control, and adherence to non-pharmaceutical interventions in an assessment of psychological patterns and participant characteristics in adults in Split, Croatia, 2021.

## Discussion

We employed PLS-SEM for measurement of a reflective model of adherence to NPIs in accordance with NPT. PLS-SEM reflective model assessment provides two-group specific factors in inverse relationships with excellent goodness-of-fit that facilitate normalization of NPIs in everyday life. Significant negative covariance factors revealed an inverse relationship between the first factor; adherence to measures and internal LoC, and the second factor; young adulthood age (≤25) and highest level of education. LoC is an expected potential mechanism by which sex and belonging to coherent subgroups can produce an indirect effect on adherence to epidemiological measures.

Coherent subgroups had a more pronounced tendency toward integration of NPIs. Group factors that facilitated normalization were higher educated younger adults with a tendency toward external LoC. Personal and behavioural factors of coherent subgroups significantly influenced NPI adherence, except for MBS. Our two-factor PLS-SEM model of adherence to NPIs based on NPT served as a more theoretical and quantitative alternative to a biomedical model that has been used by physicians and mental health professionals in planning public health interventions.

Identifying characteristics and psychological patterns of coherent subgroups influencing adherence to NPIs is crucial for public health. Understanding these factors enables officials to enhance compliance by customizing interventions for coherent social subgroups. This knowledge also aids in coordinating efforts to manage adherence to NPIs during current and future pandemics.

### Comparison to previous studies

There are only few studies that confirm that highly educated young people were more likely to adhere to NPIs showing their confidence in evidence-based COVID-19 information.^23^ Shitu and colleagues^38^ similarly determined the relationship between psychological measures and adherence to COVID-19 preventable behaviour and found that students’ self-perceived ability to complete tasks was a significant predictor of preventable behaviour.^39^ Another study found that students’ perception of COVID-19 severity correlated with compliance to preventive measures.^38^ Accordingly, our comparison of characteristics and psychological patterns of behaviour, found that medical students and people with substance use disorders significantly differed in explanatory characteristics from all groups. The variances show that it is justified to treat each group as a homogeneous target group in settings similar to this study.

### Implications for public health communication during pandemics

We assumed that knowledge management and capacity for knowledge translation (development, synthesis, contextualization and adaptation) (see Supplementary References) of medical students was a key factor contributing to decision making related to NPI adherence. Higher levels of self-efficacy, perceived severity of illness and external LoC in regard to medical professionals were all positively associated with plans to take recommended precautions. Knowing that the LoC of coherent social subgroups could be useful for public health decisions, planning preventive measures targeting social and behavioural factors may be feasible.^25^ LoC in the sense of controlling your own behaviour is not static: as people age they experience changes in the orientation of LoC so excessive that the conviction that individuals have control of events around them can be more harmful than beneficial.^21^

Paradoxically, the other coherent group comprising people with substance use disorders was more prone to normalization and acceptance of NPIs. One might think that the contextual framework (including criteria in outpatient treatment: no symptoms of COVID-19 following preventive measures) and/or social desirability bias influenced the results. However, Pooler et al. (see Supplementary References) reported on cohesion and recovery outcomes in people with substance use disorder undergoing treatment and their findings supported some observed behavioural patterns. Their findings additionally defined the latent construct of collective efficacy: significant higher interactions and group cohesion, and the interaction of sex and people with substance use disorders on measures of social support and self-efficacy.

Social–moral behaviour had no significant effect on adherence to NPIs. Participants, irrespective of sex, age, education and subgroup, who adhered or did not adhere to NPIs were equally competent in judging social and moral dilemmas in the MBS. These results suggest that participants’ behaviour included a social–moral value, demonstrating a willingness to consider aspects and consequences of moral conflicts involved in adherence.

In this study, we identified 21% of SDC residents (*n* = 137/656) exhibiting low compliance or ambivalent behaviour toward COVID-19 NPIs. These participants mostly had less than a master’s degree, were aged >25, ≤35 and >45 years, and exhibited internal LoC. These findings emphasize the need for specific strategies based on NPT to encourage behaviour change in groups with low compliance toward NPIs. Motivational interviews with mental health specialists could help increase motivation in patients to change long-standing detrimental behaviours. A recent study showed that internal LoC was an excellent predictor of vaccination when recommended by physicians during face-to-face communication (see Supplementary References). Further, patients with internal LoC can create the illusion that they are in control of their own health.

### Limitations

Our model has limitations that present opportunities for future studies. We encountered a problem known as Bernoulli trials inherent in binomial probability as we considered two outcomes for simplicity; successful or failed adherence to NPIs. We also assumed constant adherence throughout the pandemic, neglecting variation during peaks. The sample, drawn from residents in one city, may introduce bias from socially desirable responses, unaccounted factors and limited representativeness. The cross-sectional design prevents inferring causation between model factors. Future studies could explore these aspects more comprehensively.

## Supplementary Material

ckae085_Supplementary_Data

## Data Availability

The data underlying this article will be shared on reasonable request to the corresponding author. Key points Few studies assessed the relationship between adherence to non-pharmaceutical interventions (NPIs) for COVID-19 and psychological factors regarding human behaviour that could influence adherence. Age, educational level and external locus of control or low degree of control over one’s life were associated with adherence to NPIs. When creating NPIs for COVID-19 in small urban cities, public health officials should consider that preventive strategies may be more effective in highly educated adults younger than 25 years of age.
