# Supplementary material for: The reflective measurement model of adherence to non-pharmaceutical interventions (NPIs) in accordance with normalization process theory (NPT) in coherent and convenient social subgroups: PLS-SEM analysis
Source: Eur J Public Health. 2024 May 9;34(5):902–7. doi: 10.1093/eurpub/ckae085 (PMC11430931; doi:10.1093/eurpub/ckae085)
Supplement: ckae085_Supplementary_Data [file ckae085_supplementary_data.zip › ckae085_Supplementary_Data/ejph-2023-10-om-0557-File002.docx]

*Supplementary Table 1 caption*: Psychological patterns of behaviour and adherence to non-pharmaceutical interventions (NPIs) by sex, age, education, group, locus of control, and moral behavior scale in adults in Split Croatia, 2021.

*Alt text*: Table displaying the participants’ locus of control and moral behaviour and adherence to non-pharmaceutical interventions (NPIs) according to their characteristics in Split Croatia, 2021.

| **Behavior** | **Adherence to NPIs** | **Characteristics** | **N (%)** | **Median (95% CI)** |
| --- | --- | --- | --- | --- |
| Locus Control (LoC)^a^ | Adherence | Male | 206 (31.4) | 11.0 (10.5 - 11.5) |
|  |  | Female | 313 (47.7) | 12.0 (12.2 - 12.9) |
|  | Non adherence | Male | 67 (10.2) | 10.0 (9.3 - 10.6) |
|  |  | Female | 70 (10.7) | 12.0 (10.5 - 12.2) |
| Moral Behaviour (MBS)^b^ | Adherence | Male | 206 (31.4) | 42.0 (40.7 - 42.6) |
|  |  | Female | 313 (47.7) | 44.0 (42.9 - 44.3) |
|  | Non adherence | Male | 67 (10.2) | 44.0 (40.2 - 44.3) |
|  |  | Female | 70 (10.7) | 44.0 (41.4 - 44.8) |
| Locus Control (LoC) | Adherence | age ≤ 25 | 240 (36.6) | 13.0 (12.2 - 13.2) |
|  |  | 25 < age ≤ 35 | 75 (11.4) | 11.0 (10.5 - 12.1) |
|  |  | 35 < age ≤ 45 | 109 (16.6) | 11.0 (10.5 - 11.7) |
|  |  | age > 45 | 95 (14.5) | 12.0 (10.8 - 12.1) |
|  | Non adherence | age ≤ 25 | 24 (3.7) | 12.0 (10.8 - 13.2) |
|  |  | 25 < age ≤ 35 | 29 (4.4) | 9.0 (8.4 - 10.4) |
|  |  | 35 < age ≤ 45 | 40 (6.1) | 12.0 (10.2 - 12.4) |
|  |  | age > 45 | 44 (6.7) | 10.0 (9.2 - 11.0) |
| Moral Behaviour (MBS) | Adherence | age ≤ 25 | 240 (36.6) | 42.0 (40.6 - 42.1) |
|  |  | 25 < age ≤ 35 | 75 (11.4) | 41.0 (40.5 - 43.0) |
|  |  | 35 < age ≤ 45 | 109 (16.6) | 44.0 (42.7 - 45.2) |
|  |  | age > 45 | 95 (14.5) | 47.0 (44.4- 47.2) |
|  | Non adherence | age ≤ 25 | 24 (3.7) | 38.0 (34.6 - 40.5) |
|  |  | 25 < age ≤ 35 | 29 (4.4) | 40.0 (37.6 - 43.8) |
|  |  | 35 < age ≤ 45 | 40 (6.1) | 44.0 (41.4 - 45.4) |
|  |  | age > 45 | 44 (6.7) | 47.0 (44.0 - 48.3) |
| Locus Control (LoC) | Adherence | Elementary school completion | 11 (1.7) | 12.0 (9.5 - 14.3) |
|  |  | High school completion | 158 (24.1) | 11.0 (10.8 - 11.8) |
|  |  | Bachelor’s degree | 48 (7.3) | 10.0 (9.7 - 11.5) |
|  |  | Master’s degree | 302 (46.0) | 12.0 (12.0 - 12.9) |
|  | Non adherence | Elementary school completion | 12 (1.8) | 11.0 (8.7 - 12.6) |
|  |  | High school completion | 65 (9.9) | 10.0 (10.1 - 11.5) |
|  |  | Bachelor’s degree | 15 (2.3) | 10.0 (7.6 - 11.8) |
|  |  | Master’s degree | 45 (6.9) | 11.0 (9.7 - 11.7) |
| Moral Behaviour (MBS) | Adherence | Elementary school completion | 11 (1.7) | 43.0 (39.4 - 44.4) |
|  |  | High school completion | 158 (24.1) | 44.0 (42.8 - 45.1) |
|  |  | Bachelor’s degree | 48 (7.3) | 44.5 (41.9 - 45.8) |
|  |  | Master’s degree | 302 (46.0) | 42.5 (41.4 - 42.8) |
|  | Non adherence | Elementary school completion | 12 (1.8) | 42.0 (37.2 - 45.3) |
|  |  | High school completion | 65 (9.9) | 42.0 (39.4 - 43.5) |
|  |  | Bachelor’s degree | 15 (2.3) | 48.0 (41.5 - 50.0) |
|  |  | Master’s degree | 45 (6.9) | 45.0 (41.8 - 45.9) |
| Locus Control (LoC) | Adherence | COVID-19 infection suspected | 155 (23.6) | 11.0 (10.5 - 11.4) |
|  |  | Travelers | 87 (13.3) | 11.0 (10.2 - 11.5) |
|  |  | People with substance abuse disorders | 66 (10.1) | 12.0 (10.8 - 12.7) |
|  |  | Medical students | 211 (32.2) | 13.0 (12.7 - 13.7) |
|  | Non adherence | COVID-19 infection suspected | 59 (9.0) | 11.0 (10.3 - 11.7) |
|  |  | Travelers | 15 (2.3) | 10.0 (7.8 - 11.0) |
|  |  | People with substance abuse disorders | 57 (8.7) | 10.0 (9.3 - 11.0) |
|  |  | Medical students | 6 (1.0) | 15.0 (12.9 - 17.5) |
| Moral Behaviour (MBS) | Adherence | COVID-19 infection suspected | 155 (23.6) | 43.0 (41.5 - 44.0) |
|  |  | Travelers | 87 (13.3) | 44.0 (43.5 - 46.1) |
|  |  | People with substance abuse disorders | 66 (10.1) | 45.0 (43.2 - 46.3) |
|  |  | Medical students | 211 (32.2) | 42.0 (40.7 - 42.2) |
|  | Non adherence | COVID-19 infection suspected | 59 (9.0) | 43.0 (38.9 - 43.5) |
|  |  | Travelers | 15 (2.3) | 48.0 (44.7 - 50.0) |
|  |  | People with substance abuse disorders | 57 (8.7) | 44.0 (41.3 - 45.0) |
|  |  | Medical students | 6 (1.0) | 41.5 (36.7 - 46.0) |

*Note.* NPIs = non-pharmaceutical interventions, CI = confidence interval.
