# Supplementary material for: The reflective measurement model of adherence to non-pharmaceutical interventions (NPIs) in accordance with normalization process theory (NPT) in coherent and convenient social subgroups: PLS-SEM analysis
Source: Eur J Public Health. 2024 May 9;34(5):902–7. doi: 10.1093/eurpub/ckae085 (PMC11430931; doi:10.1093/eurpub/ckae085)
Supplement: ckae085_Supplementary_Data [file ckae085_supplementary_data.zip › ckae085_Supplementary_Data/ejph-2023-10-om-0557-File003.docx]

*Supplementary Table 2 caption*: Comparison of the variance of adherence to non-pharmaceutical interventions (NPIs) according to adult participants' characteristics and personal behaviour in Split, Croatia in 2021.

*Alt text*: Table showing the comparisons of the variances of adherence to non-pharmaceutical interventions (NPIs) according to adult participants' characteristics and personal behaviour in Split, Croatia in 2021.

| **Characteristics** | **χ²** | **df** | **P-value^a^** | **η2^b^** |
| --- | --- | --- | --- | --- |
| Sex | 3,781 | 1 | 0.05 | 0.00577 |
| Age | 33,565 | 1 | < .001^c^ | 0.05124 |
| Education | 32,796 | 1 | < .001^c^ | 0.05007 |
| Locus Control (LoC) | 12,193 | 1 | < .001^c^ | 0.01862 |
| Moral Behaviour (MBS) | 0.138 | 1 | 0.71 | 2.10e-4 |

^a^Kruskal-Wallis test with a P-value set at <.05.

^b^η2 - effect size reflects the percentage of the variance in the dependent variable explained by the independent variables in the sample.

^c^P < .001 vs. sex and MBS (Kruskal-Wallis test with Dwass-Steel-Critchlow-Fligner post hoc test).
