# Supplementary material for: The reflective measurement model of adherence to non-pharmaceutical interventions (NPIs) in accordance with normalization process theory (NPT) in coherent and convenient social subgroups: PLS-SEM analysis
Source: Eur J Public Health. 2024 May 9;34(5):902–7. doi: 10.1093/eurpub/ckae085 (PMC11430931; doi:10.1093/eurpub/ckae085)
Supplement: ckae085_Supplementary_Data [file ckae085_supplementary_data.zip › ckae085_Supplementary_Data/ejph-2023-10-om-0557-File004.docx]

*Supplementary Table 3 caption*: Comparison of the variance of groups by personal behaviour determinants in the assessment of psychological patterns, participant characteristics, and non-pharmaceutical interventions in adults in Split, Croatia in 2021.

*Alt text*: Table showing the comparison of the sample variances of the groups of participants according to their psychological patterns, characteristics, and adherence to non-pharmaceutical interventions.

| **Characteristics** | **χ²** | **df** | **P-value^a^** | **η2^b^** |
| --- | --- | --- | --- | --- |
| Sex | 114.9 | 3 | < .001^c^ | 0.1754 |
| Age | 357.1 | 3 | < .001^c^ | 0.5452 |
| Education | 353.0 | 3 | < .001^c^ | 0.5389 |
| Locus Control (LoC) | 61.5 | 3 | < .001^c^ | 0.0939 |
| Moral Behaviour (MBS) | 26.2 | 3 | < .001^c^ | 0.0400 |
| Adherence to NPIs | 99.4 | 3 | < .001^c^ | 0.1517 |

*Note.* NPIs = non-pharmaceutical interventions.

a Kruskal-Wallis test with a significance level set at P<0.05.

b η2 - effect size and reflects the percentage of the variance in the dependent variable explained by the independent variables in a sample.

c P < .001 vs. sex, age, education, LoC, MBS, and adherence to NPIs (Kruskal-Wallis test with Dwass-Steel-Critchlow-Fligner post hoc test).
