# Supplementary material for: The reflective measurement model of adherence to non-pharmaceutical interventions (NPIs) in accordance with normalization process theory (NPT) in coherent and convenient social subgroups: PLS-SEM analysis
Source: Eur J Public Health. 2024 May 9;34(5):902–7. doi: 10.1093/eurpub/ckae085 (PMC11430931; doi:10.1093/eurpub/ckae085)
Supplement: ckae085_Supplementary_Data [file ckae085_supplementary_data.zip › ckae085_Supplementary_Data/ejph-2023-10-om-0557-File005.docx]

*Supplementary Table 4 caption*: Dwass-Steel-Critchlow-Fligner pairwise comparisons of variances according to sex, age, education, LoC and MBS in the assessment of psychological patterns, participant characteristics, and non-pharmaceutical interventions in adults in Split, Croatia in 2021.

*Alt text*: Table showing the Dwass-Steel-Critchlow-Fligner post hoc comparisons of the variances of sex, age, education, LoC and MBS in assessing psychological patterns, participant characteristics, and non-pharmaceutical interventions in adults in Split, Croatia in 2021.

| Characteristic |  |  |
| --- | --- | --- |
| **Sex** | **W** | **P-value** |
| COVID-19 infection suspected - Travellers | -5.41 | < .001 |
| COVID-19 infection suspected - People with substance abuse disorders | -8.82 | < .001 |
| COVID-19 infection suspected - Medical students | 6.34 | < .001 |
| Travellers - People with substance abuse disorders | -2.79 | 0.199 |
| Travellers - Medical students | 10.68 | < .001 |
| People with substance abuse disorders - Medical students | 14.09 | < .001 |
| **Age** |  |  |
| COVID-19 infection suspected - Travellers | -3.30 | 0.090 |
| COVID-19 infection suspected - People with substance abuse disorders | -0.02 | 1,000 |
| COVID-19 infection suspected - Medical students | -24.01 | < .001 |
| Travellers - People with substance abuse disorders | 3.38 | 0.079 |
| Travellers - Medical students | -20.29 | < .001 |
| People with substance abuse disorders - Medical students | -23.04 | < .001 |
| **Education level** |  |  |
| COVID-19 infection suspected - Travellers | 4.43 | 0.009 |
| COVID-19 infection suspected - People with substance abuse disorders | -12.09 | < .001 |
| COVID-19 infection suspected - Medical students | 19.99 | < .001 |
| Travellers - People with substance abuse disorders | -13.58 | < .001 |
| Travellers - Medical students | 16.17 | < .001 |
| People with substance abuse disorders - Medical students | 24.95 | < .001 |
| **LoC** |  |  |
| COVID-19 infection suspected - Travellers | -1.23 | 0.821 |
| COVID-19 infection suspected - People with substance abuse disorders | 0.35 | 0.995 |
| COVID-19 infection suspected - Medical students | 9.64 | < .001 |
| Travellers - People with substance abuse disorders | 1.32 | 0.788 |
| Travellers - Medical students | 8.4 | < .001 |
| People with substance abuse disorders - Medical students | 7.11 | < .001 |
| **MBS** |  |  |
| COVID-19 infection suspected - Travellers | 3.75 | 0.040 |
| COVID-19 infection suspected - People with substance abuse disorders | 1.96 | 0.506 |
| COVID-19 infection suspected - Medical students | -2.86 | 0.180 |
| Travellers - People with substance abuse disorders | -1.89 | 0.538 |
| Travellers - Medical students | -7.01 | < .001 |
| People with substance abuse disorders - Medical students | -5.07 | 0.002 |
| **Adherence to NPIs** |  |  |
| COVID-19 infection suspected - Travellers | -3.56 | 0.057 |
| COVID-19 infection suspected - People with substance abuse disorders | 4.93 | 0.003 |
| COVID-19 infection suspected - Medical students | -10.16 | < .001 |
| Travellers - People with substance abuse disorders | 7.15 | < .001 |
| Travellers - Medical students | -5.66 | < .001 |
| People with substance abuse disorders - Medical students | -14.03 | < .001 |

*Note.* LoC = locus of control; MBS = moral behavior scale; NPIs = non-pharmaceutical interventions.
