# Supplementary material for: The reflective measurement model of adherence to non-pharmaceutical interventions (NPIs) in accordance with normalization process theory (NPT) in coherent and convenient social subgroups: PLS-SEM analysis
Source: Eur J Public Health. 2024 May 9;34(5):902–7. doi: 10.1093/eurpub/ckae085 (PMC11430931; doi:10.1093/eurpub/ckae085)
Supplement: ckae085_Supplementary_Data [file ckae085_supplementary_data.zip › ckae085_Supplementary_Data/ejph-2023-10-om-0557-File006.docx]

| 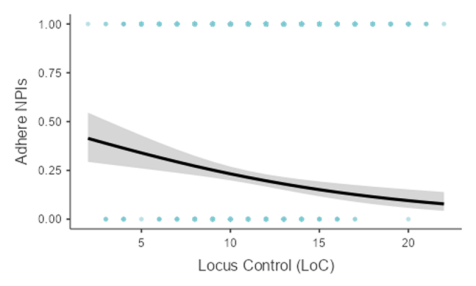 | 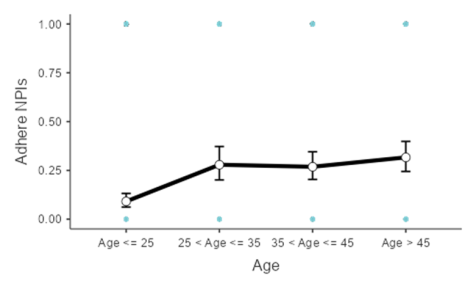 | 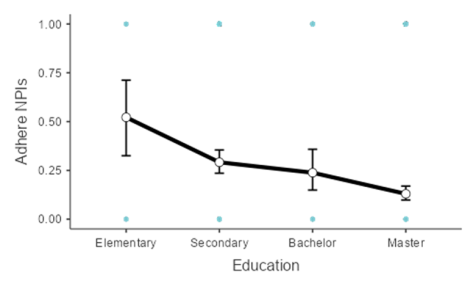 |
| --- | --- | --- |
| 1. Adherence by locus of control | 1. Adherence by age | 1. Adherence by education |

*Supplementary Figure 1 caption*: Probability of adherence (0) or non-adherence (1) to non-pharmaceutical interventions (NPIs) as a function of characteristics (age and education) and psychological patterns of human behaviour (locus of control) in the assessment of psychological patterns, participant characteristics, and NPIs in adults in Split, Croatia in 2021.

*Alt text*: Line graphs displaying the likelihood of individuals’ adherence to non-pharmaceutical interventions according to behaviour (locus of control), age, and education in Split, Croatia in 2021.
