# Supplementary material for: The reflective measurement model of adherence to non-pharmaceutical interventions (NPIs) in accordance with normalization process theory (NPT) in coherent and convenient social subgroups: PLS-SEM analysis
Source: Eur J Public Health. 2024 May 9;34(5):902–7. doi: 10.1093/eurpub/ckae085 (PMC11430931; doi:10.1093/eurpub/ckae085)
Supplement: ckae085_Supplementary_Data [file ckae085_supplementary_data.zip › ckae085_Supplementary_Data/ejph-2023-10-om-0557-File007.docx]

|  |  |
| --- | --- |
| Model 1. | Model 2. |
|  |  |
| Model 3. | Model 4. |

*Supplementary Figure 2 caption*: Comprehensive evaluation of the selection criteria of four models in the assessment of psychological patterns, participant characteristics, and non-pharmaceutical interventions in adults in Split, Croatia, 2021.

*Alt text*: Figure showing models generated by partial least squares structural equation modeling (PLS-SEM) to assess locus of control, participant characteristics, and non-pharmaceutical interventions in adults in Split, Croatia, 2021.
