# Supplementary material for: The reflective measurement model of adherence to non-pharmaceutical interventions (NPIs) in accordance with normalization process theory (NPT) in coherent and convenient social subgroups: PLS-SEM analysis
Source: Eur J Public Health. 2024 May 9;34(5):902–7. doi: 10.1093/eurpub/ckae085 (PMC11430931; doi:10.1093/eurpub/ckae085)
Supplement: ckae085_Supplementary_Data [file ckae085_supplementary_data.zip › ckae085_Supplementary_Data/ejph-2023-10-om-0557-File008.docx]

**Supplementary Appendix A: Participant survey that collected sociodemographic data, and contained the Moral Behavior Scale* and Rotter's Locus of Control Scale^†^.**

*The Moral Behavior Scale: Compliance to explicit social and moral norms was evaluated using 15 items with a minimum 15 and maximum 60 points.

See citation: Mendez MF, Anderson E, Shapira JS. An investigation of moral judgement in frontotemporal dementia. *Cogn Behav Neurol* 2005;18(4):193-197. doi: 10.1097/01.wnn.0000191292.17964.bb.

^†^Rotter's Locus of Control Scale: Contains 29 statement pairs, whereby participants choose the answers that better reflect their beliefs that their life is controlled by themselves or other factors. The total minimum points is 0 while the maximum is 23 points.

See citation: Rotter J. Generalized expectancies for internal versus external control of reinforcement. *Psychol Monogr* 1966;80(1):1–28.

We're asking for your participation in this anonymous survey, to get a better understanding of reasons people adhere to health-related measures during the COVID-19 pandemic.

The questionnaire examines your experiences and attitudes related to a number of preventive measures introduced during the COVID-19 pandemic. Please answer the questions honestly as the survey is anonymous.

Your identity as well as your responses will remain anonymous at all times. Your personal data will be processed electronically, and the researchers will handle data collection and analysis in compliance with regulations to protect your personal data.

By completing the survey, you agree to participate in the research, and for any additional questions, feel free to contact us.

Thank you in advance for your time and cooperation!

* Indicates a required question

Personal characteristics

1. Sex?*

__M

__F

__Unwilling to disclose

1. Year of birth: _____________
2. At which school are you a student:

__School of Medicine

__School of Humanities

__School of Natural Sciences

1. Choose your study year

__1^st^

__2^nd^

__3^rd^

__4^th^

__5^th^

_6^th^

1. Select which university you attend:

__University of Split

__University of Zagreb

__University of Rijeka

__University of Osijek

__Other

1. Where is your temporary residence?:

______

1. Where is your permanent residence?:

______

1. Have you recovered from a COVID-19 infection?

__Yes

__No

1. How long did you have symptoms after getting better?

__3-5 days

__6-9 days

__10 or more days

__ Did not have symptoms

1. Registered number of people you have been in close contact with:

__1-4 people

__5-9 people

__10 or more

__No close contact with anyone

1. Relationship with the people you have been in close contact with:

__Partner

__Family member

__Friend

__Acquaintances

__Unknown

1. Are you a smoker?

__No

__Occasionally

__1/2 pack a day

__1 pack a day

__More than 1 pack a day

1. How frequently do you drink alcoholic beverages?

__Never

__Daily

__Weekends

__1-2 times a month

1. How frequently do you take drugs?

__Never

__Daily

__Weekend

__1-2 times a month

1. If you take drugs, which drug(s)?

_____

1. How frequently do you gamble?

__Never

__Daily

__Weekend

__1-2 times a month

1. Do you take psychotropic drugs?

__No

__ Anxiolytics

__ Antidepressants

__ Psychotics

__ Mood stabilizers

1. If you take psychotropic drugs, do you take them following your doctor’s recommendations?

__Yes

__No

1. If you take psychotropic drugs, how long have you been abstinent?

__1 month

__2-3 months

__4-12 months

__1 year or more

1. How did the "lockdown" affect your financial situation?

__No income at all

__Decreased income

__Not enough for basic living expenses

__Needed government welfare support

__Income remained unchanged or increased

1. Did you consider quitting school because of your financial situation?

__Yes

__No

1. Do you think other people are going through the same thing as you?

__ I don't think at all about how others are doing

__ It's good for me how it is for others

__ I have it worse than others

__ What is happening to me is incomparably more difficult than to anyone else

Evaluate the following behaviors below:

|  | Not a mistake | Slight mistake | Moderate mistake | Serious mistake |
| --- | --- | --- | --- | --- |
| 1. Not keeping promises. |  |  |  |  |
| 2. Taking the last seat in a crowded bus. |  |  |  |  |
| 3. Selling someone a defective car. |  |  |  |  |
| 4. Drinking and driving. |  |  |  |  |
| 5. Cutting in line when in a hurry. |  |  |  |  |
| 6. Not donating blood. |  |  |  |  |
| 7. Being mean to someone just because you don't like them. |  |  |  |  |
| 8. Slightly lying to minimize harm. |  |  |  |  |
| 9. Driving the homeless out of your neighborhood. |  |  |  |  |
| 10. Not helping someone pick up papers they dropped. |  |  |  |  |
| 11. Keeping the change from a purchase in a store. |  |  |  |  |
| 12. Not offering help after an accident. |  |  |  |  |
| 13. Not providing food for a hungry stranger. |  |  |  |  |
| 14. Not voting in minor elections. |  |  |  |  |
| 15. Keeping money found on the ground. |  |  |  |  |

Choose one of the statements from each pair that you agree with the most:

1. A. Children get into trouble because their parents punish them too much.

B. Most children’s problems today arise because their parents are too gentle on them.

1. A. Many unfortunate things in people's lives are partly due to bad luck.

B. People's misfortunes result from the mistakes they make.

1. A. One of the main reasons we have wars is because people are not interested in politics.

B. There will always be wars, no matter how hard people try to prevent them.

1. A. In the long run, people realize the respect they deserve in this world

B. Unfortunately, individual values often go unnoticed no matter how hard we try

1. A. The idea that teachers are unfair to students is nonsense.

B. Most students do not realize the extent to which their grades are influenced by random events.

1. A. Without necessary harshness, a person cannot be an effective leader.

B. Capable people who fail to become leaders have not taken advantage of their opportunities.

1. A. No matter how hard you try around some people, they just don't like you.

B. People who cannot teach others to love them do not understand how to treat others.

1. A. Heredity plays a large role in determining one's personality

B. Heredity is the experience in life that determines who you are.

1. A. I have often predicted what I thought would happen.

B. It would never work out for me if I relied on fate, it would always be better if I made decisions rationally

1. A. It is rare for a well-prepared student to complain about a test.

B. Many times exam questions are not related to practical work and learning is useless.

1. A. Success is the result of hard work, luck has little or nothing to do with it.

B. Getting a good job depends mostly on being in the right place at the right time.

1. A. The average citizen can have an influence on government decisions.

B. This world is run by a few people in power, and there's not much the little people can do about it.

1. A. When I set a plan for myself, I am almost certain that I can do it.

B. It is not always wise to plan too far in advance because many things go wrong - it is all a matter of good and bad luck.

1. A. There are certain people who are just not good.

B. There is something good in all people.

1. A. In my case, getting what I want has little or nothing to do with happiness.

B. Many times we could decide what to do by flipping a coin.

1. A. Who gets to be the boss often depends on who was lucky enough to be in the right place at the right time.

B. Getting people to do the right thing depends on ability. Luck has little or nothing to do with it.

1. A. Considering what is happening in the world, most of us are victims of forces that we can neither understand nor control.

B. Through active participation in political and social events, people can control world events.

1. A. Most people do not realize the extent to which their lives are controlled by random events.

B. Nothing is as valuable as "happiness".

1. A. One should always be willing to admit mistakes.

B. It is usually best to cover up your mistakes.

1. A. It is difficult to know if a person loves you or not.

B. How many friends you have depends on how nice a person you are.

1. A. In the long run, the bad things that happen to us are balanced with the good.

B. Most accidents are the result of lack of ability, ignorance, laziness, or all three combined.

1. A. With enough effort we can eradicate political corruption.

B. People find it difficult to control what politicians do while in office.

1. A. Sometimes I can't understand how teachers give the grades they do.

B. There is a direct relationship between how hard I studied and the grades I got.

1. A. A good leader expects people to decide for themselves what they should do.

B. A good leader clearly tells everyone what their jobs are.

1. A. Many times I feel that I have little influence over the things that happen to me.

B. It is impossible for me to believe that chance or luck plays an important role in my life.

1. A. People are lonely because they don't even try to be friendly.

B. There's no point in trying too hard to please people, if they like you, they like you.

1. A. Too much emphasis is placed on sports in high school.

B. Team sports are a great way to build character.

1. A. What happens to me is my own doing.

B. Sometimes I feel like I don't have enough control over the direction my life is going.

1. A. Most of the time I cannot understand why politicians behave the way they do.

B. In the long run, people are responsible for a bad government at the national as well as the local level.
